# Editorial: Understanding, assessing, and guiding adaptations in public health and health systems interventions: current and future directions

**DOI:** 10.3389/fpubh.2023.1228437

**Published:** 2023-06-27

**Authors:** Roman Aydiko Ayele, Borsika Adrienn Rabin, Marina McCreight, Catherine Battaglia

**Affiliations:** ^1^Seattle-Denver Center of Innovation, VA Eastern Colorado Health Care System, Denver, CO, United States; ^2^School of Medicine, University of Colorado Anschutz Medical Campus, Aurora, CO, United States; ^3^Altman Clinical and Translational Research Institute, School of Medicine, University of California, San Diego, La Jolla, CA, United States; ^4^Herbert Wertheim School of Public Health and Human Longevity Science, University of California, San Diego, La Jolla, CA, United States; ^5^Colorado School of Public Health, University of Colorado Anschutz Medical Campus, Aurora, CO, United States

**Keywords:** adaptations, implementation science, implementation strategies, pragmatic approach, adaptation context

There is a growing agreement that adaptations or changes to an intervention and implementation strategies are inevitable to support the implementation and uptake of interventions in real world settings (1). A critical area of research is emerging in dissemination and implementation science to better understand what adaptations are made, assess reasons why and when adaptations were made, and with what impact before and during implementation of public health and health care programs (2). To answer these questions there is a need to systematically document and assess adaptations across the life cycle of a program (Tempelaar et al.). Methods are still evolving, and a range of questions remain to be studied. These questions include but are not limited to:

1. What aspects of an intervention and an implementation strategy can be adapted and to what extent and who decides these adaptations;
2. What are pragmatic approaches to documenting adaptations;
3. How do we assess the impact of adaptations on implementation and effectiveness outcomes;
4. How can we use real-time information about adaptations to guide improvement;
5. How do contextual factors influence these issues; and
6. How can we meaningfully involve community and implementation partners in these activities.

To advance the field and address these adaptation issues, we initiated the Research Topic on Understanding, Assessing, and Guiding Adaptations in Public Health and Health Systems Interventions: Current and Future Directions. The primary goal of the Research Topic was to highlight cutting-edge work on understanding, assessing, and guiding adaptations, and explore future directions. We indicated interest in work that addressed adaptations in a variety of contexts and described innovative research that demonstrated and highlighted opportunities for future investigation and provided a multi-dimensional perspective as well as work from across the world, with a focus on original research.

We are pleased to present a collection of 21 papers in this Research Topic that delve into the complexities of adapting interventions in the public health and health systems domain. The papers presented here provide a comprehensive overview of the current state of research on this topic, along with insights into future directions for research and practice. In addition to this editorial, a perspective was also provided by Dr. David Chambers to synthesize key lessons learned from these 21 papers and propose next steps and directions for the field (Chambers).

The studies described in the included papers took place in diverse geographical locations and settings and focused on a variety of health topics and populations. We also noted a diversity in terms of the adaptation topics addressed by the papers.

While most papers described studies conducted in the United States, additional geographical locations included Canada (Tempelaar et al.) Chile (Le et al.) and Sweden (Pettersson et al.).

Health topics were very diverse and ranged from focus on increasing breastfeeding (Glasgow et al.), a general consideration for public health emergencies such as the COVID-19 pandemic (Eisman et al.) social risk screening and referral (Cohen et al.), providing multidisciplinary care for individuals with first episode psychosis (Le et al.), support tool for reducing cardiovascular risk in women Veterans (Brunner et al.) clinical interventions with a focus on mental health (Stirman et al.).

When exploring settings across these papers, there were 13 that were conducted in clinical settings, one in a community setting, four included both clinical and community settings, while three papers focused on general population. Most common clinical settings included the VA—largest integrated health care system in the United States.

Papers also described a wide variety of priority populations ranging from children, adolescents, caregivers, women, Latinx community, as well as various clinicians, administrators, and policymakers. Concerns for health equity were mentioned or key focus of a number of the included papers. Specifically, Williamson et al. described plans to adapt a model to evaluate implementation of a sleep intervention with adolescents of minoritized backgrounds, while Kamen et al. described adapting a cultural humility training program in clinical oncology practices.

We found that 17 of the included papers described how they identified and documented adaptations and three focused on assessing the impact of adaptations on implementation outcomes, including economic implication of adaptations (Rhodes et al.). Multiple papers used specific adaptation theories, models, and frameworks (TMF) to guide the documentation and impact assessment of adaptations. The most commonly used TMF was the expanded framework for reporting adaptations and modifications to evidence-based interventions (FRAME) and its implementation strategy focused variation, the FRAME-IS (*n* = 10 for FRAME and FRAME-IS).

Examples of additional models included are the model for adaptation design and impact (MADI), the Reach, Effectiveness, Adoption, Implementation and Maintenance framework and its contextually expanded version the Practical, Robust Implementation and Sustainability Model (RE-AIM/PRISM), the ADAPT-ITT, and the Consolidated Framework for Implementation Research (CFIR).

Three key themes emerged from these papers: First, the importance of understanding the contextual factors that influence the success of intervention adaptations. Several papers examine the role of culture, policy, and partner engagement in shaping the adaptation and implementation of interventions. For example, Kamen et al. highlights the importance of cultural humility training for oncology providers and staff to address the political and social context specific practice environments and advocate for broader institutional culture chance to achieve responsiveness to sexual and gender minority health needs.

The second theme that emerged is the need for effective tools and strategies to assess the fidelity and effectiveness of interventions. Several papers present innovative approaches for evaluating the outcomes of interventions, such as the use of realist evaluation frameworks or the integration of implementation science principles into evaluation design. These approaches offer valuable insights into the complex interplay between intervention components, implementation processes, and outcomes.

Third, several papers highlight the importance of guiding interventions through ongoing feedback and adaptation. For example, McNeal et al. described multi-methods evaluation of an evidence-based training program using real-time stakeholder feedback to guide intervention translation from research to practice settings. This approach underscores the importance of collaboration and ongoing communication with partners to ensure the effectiveness of interventions.

Key, ongoing challenges for the field is to better identify what counts as an adaptation, identify what methods or combination of methods might be optimal to document adaptations—considering both comprehensiveness and pragmatism, and to find better ways to document the impact of adaptations. In this collection there were only three papers that attempted to capture the impact of adaptations. More systematic use of models can also support cross-project comparisons.

## Author contributions

All authors were involved in the conceptualization of the editorial, drafting, contributed to the final writing process, and approved the submitted version.
